# Editorial: Engineering biomaterials for vascular embolization

**DOI:** 10.3389/fbioe.2023.1186609

**Published:** 2023-03-28

**Authors:** Qun Tang

**Affiliations:** ^1^ Jiangxi Provincial Key Laboratory of Preventive Medicine, School of Public Health, Nanchang University, Nanchang, China; ^2^ Institute for Advanced Study, Nanchang University, Nanchang, China

**Keywords:** editorial, biomaterials, vascular embolization, liver cancer, microsphere

The Research Topic *Engineering biomaterials for vascular embolization* in Frontiers in Bioengineering and Biotechnology aims to present “bench-to-bedside” contributions focusing on recent advances in this important area. The annual industrial output value of embolic biomaterials is worth of millions of US dollars and the market is still growing very fast. In the Frontier area, an increasing number of new materials, technologies, and therapeutic strategies are emerging. Lots of clinical and fundamental advances have been made recently in this clinically important and scientifically challenging research area, including shape-memory polymeric coils, *in situ* polymerization or gelling systems, and radiomicrospheres. These new concepts are gradually being commercialized.

This Research Topic comprises five papers from excellent researchers around the world, including the United States, China, and the United Kingdom. A wide range of basic and clinical Research Topic is covered, including “Material characterization of GPX^®^: a versatile *in situ* solidifying embolic platform technology” (Stewart et al.), “Hepatic arterial infusion chemotherapy versus transarterial chemoembolization for unresectable hepatocellular carcinoma: a systematic review with meta-analysis” (Si et al.), “DEB-TACE with irinotecan versus C-TACE for unresectable intrahepatic cholangiocarcinoma: a prospective clinical study” (Wang et al.), “Clinical outcomes of vinorelbine loading CalliSpheres beads in the treatment of previously treated advanced lung cancer with progressive refractory obstructive atelectasis” (Ma et al.), and “Evaluation of the safety and efficacy of transarterial sevelamer embolization in a rabbit liver cancer model: a challenge on the size rule for vascular occlusion” (Chen et al.). Both clinicians and scientists in the lab contribute their research to this special issue due to the importance of embolization therapy as well as embolic materials.

The research article by Stewart et al. presented the *in situ* solidifying embolic technique by liquid-to-solid transition. GPX is a stable aqueous solution of oppositely charged polyelectrolytes and has an *in-situ* solidification response to ionic strength gradients at the delivery site, thereby iodine-based contrast agent can be temporarily conjugated in the framework of GPX, gradually dissipating within hours. Moreover, the anticancer doxorubicin release profile was linear over 90 days. The releasing kinetic behaves as that of DOX-eluting embolic beads.

Yun Ma and Nigel Heaton from King’s College Hospital conducted a meta-analysis of patients treated with HAIC or TACE to look for differences in survival, adverse events, mortality, and downstaging. This is a long-disputed issue: which of HAIC or TACE is better? A total of 1,060 patients (TACE group, 534; HAIC group, 526) were screened, and it was summarized that patients with unresectable HCC could potentially benefit more from HAIC than from standard TACE treatment; therefore, they recommended the re-evaluation of HAIC as a treatment option in intermediate and advanced HCC. We acknowledge that their analysis is correct and the conclusion is reliable; however, the more complex issue with regard to clinical indication is liver function. HAIC might not be applicable for patients with liver dysfunction due to the drug tolerance.

The article by Bing Jie and Sen Jiang et al. evaluates the clinical outcomes of vinorelbine loading CalliSpheres beads in the treatment of progressive lung cancer with refractory obstructive atelectasis. The CalliSpheres bead is one of the commercial microspheres loaded with vinorelbine, the anticancer drug approved for treating non-small cell lung cancer. A total of 20 patients were enrolled, and the primary endpoints were the objective response rate (ORR) and improvement rate of dyspnea. Bronchial arterial chemoembolization (BACE) with this microsphere was proven to be a feasible option for progressive advanced lung cancer with obstructive atelectasis after the failure of other treatments.

The research article by Hui Xie et al. delineated a prospective clinical study to compare the clinical outcome of DEB-TACE plus irinotecan with C-TACE plus irinotecan for the patients with unresectable intrahepatic cholangiocarcinoma (ICC). The primary endpoints were objective response rate (ORR) and progression-free survival (PFS) using m-RECIST to evaluate the tumors. DEB-TACE with irinotecan showed a better ORR and PFS than C-TACE with irinotecan in the treatment of unresectable ICC.

The research article by Qun Tang et al. from Nanchang University introduced a Pi-induced aggregation polymer for vascular embolization. Sevelamer is an oral phosphate binder that treats hyperphosphatemia. Sevelamer can be ground into nanosized particles, and after the absorption of Pi, their morphology varies remarkably from individual ultrafine particles to large aggregates, as phosphate ions induce adjacent crystal face to swell and merge each other. This characteristic of Pi-induced aggregation might make sevelamer a size-adaptable MP for full embolization. Normally the bead size of D-TACE has been uniformly distributed (50–300 μm, whereas the diameter of the arterial vessel is continuously shrinking from hundreds of micrometers to a few micrometers, for example, 7–9 μm for capillaries. Therefore, theoretically the beads are only adaptable to embolize partial vessels. The commercial beads’size is strictly limited to a minimum size of 40 μm, as the beads with the size smaller than 40 μm can easily be washed out and transported to the normal liver lobe or other organs, causing severe adverse events and failed embolization. Sevelamer particles are highly mobile and behave like a liquid or gelation agent; therefore, they will go through the whole blood vessel on the proximal end and penetrate deep blood capillaries. Furthermore, their aggregation is responsive to endogenous serum Pi ions, which induce them to swell and aggregate into bigger particulates, thereby occluding the tumor-feeding vessels. Eventually, Pi absorption induces sevelamer aggregation and full embolization after arterial administration.

In summary, in this fast-growing area, further interesting reports will be published soon. This Research Topic demonstrates several typical developments that could benefit further advancements in this important area.

